# Research on Loosening Fault Diagnosis Method of Escalator Drive Mainframe Anchor Bolts Based on Improved High-Strength Denoising RCDAE Model

**DOI:** 10.3390/s25175219

**Published:** 2025-08-22

**Authors:** Dongdong Chen, Minghui Chen, Binxin Lang, Xiaoqing Wang, Qiang Xu, Jiong Shen, Lihua Liang, Qin Luo

**Affiliations:** 1 Key Laboratory of Special Equipment Safety Testing Technology of Zhejiang Province, Zhejiang Academy of Special Equipment Science, Hangzhou 310020, China; chendd@zjtj.org (D.C.);; 2 College of Mechanical and Electrical Engineering, China Jiliang University, Hangzhou 310018, China; 3 College of Mechanical Engineering, Zhejiang University of Technology, Hangzhou 310023, China

**Keywords:** RCDAE, cross-attention mechanism, escalator, anchor bolts, fault diagnosis, CNN, transformer

## Abstract

To address the challenges of weak early-stage loosening fault signals and strong environmental noise interference in escalator drive mainframe anchor bolts, which hinder effective fault feature extraction, this paper proposes an improved Residual Convolutional Denoising Autoencoder (RCDAE) for signal denoising in high-intensity noise environments. The model combines DMS (Dynamically Multimodal Synergistic) loss function, the gated residual mechanism, and CNN–Transformer. The experimental results demonstrate that the proposed model achieves an average accuracy of 93.88% under noise intensities ranging from 10 dB to −10 dB, representing a 2.65% improvement over the baseline model without the improved RCDAE (91.23%). At the same time, in order to verify the generalization performance of the model, the CWRU bearing data set is used to conduct experiments under the same conditions. The experimental results show that the accuracy of the proposed model is 1.30% higher than that of the baseline model without improved RCDAE, validating the method’s significant advantages in noise suppression and feature representation. This study provides an effective solution for loosening fault diagnosis of escalator drive mainframe anchor bolts.

## 1. Introduction

Escalators, which are efficient and convenient vertical transportation systems [1,2,3], have been widely implemented in public spaces with high pedestrian traffic and dense crowds, such as transportation hubs and large-scale commercial facilities [4,5]. Due to their operational efficiency and accessibility, escalators significantly reduce traffic congestion in densely populated areas [6]. These advantages significantly improve daily commuting, making escalators the preferred vertical mobility solution for passengers [7,8,9]. However, the frequent occurrence of escalator-related safety incidents in recent years [10,11] highlights that ensuring safe operation must be the primary priority in escalator management [12,13,14].

The safe operation of escalators is critical to the operational status of their drive mainframes. Anchor bolts in the drive mainframe are highly susceptible to loosening phenomena during practical service conditions [15], which are caused by factors such as sustained high-intensity passenger foot traffic and prolonged exposure to heavy operational loads [16,17,18]. Such loosening may cause operational instability in escalators, resulting in abnormal vibrations and acoustic emissions, compromising passenger comfort [19,20,21] and potentially escalating to safety hazards. Furthermore, bolt loosening disrupts mechanical equilibrium, accelerates wear of associated components, reduces equipment lifespan, and may result in increased energy consumption.

Current escalator maintenance practices primarily use corrective maintenance strategies [22], with interventions implemented only after a fault occurs or performance degrades below acceptable thresholds. However, such post-failure approaches are inherently inefficient, costly, and have significant diagnostic latency, resulting in suboptimal resource allocation and operational redundancy [23]. Consequently, developing a robust loosening fault diagnosis method for escalator drive mainframe anchor bolts holds critical importance in advancing predictive maintenance paradigms.

Qian Shujie et al. [24] proposed an escalator vibration fault diagnosis method using the Fast Fourier Transform (FFT) algorithm. Han Pengpeng et al. [25] developed an early-stage bearing fault diagnosis approach based on Variational Mode Decomposition (VMD) and enhanced envelope spectrum analysis, which was further optimized using genetic algorithms. Zhang et al. [26] proposed a motor-bearing fault diagnosis methodology for escalators that uses envelope demodulation, spectral kurtosis for optimal bandpass filter frequency band selection, and linear prediction model-based pre-whitening processing to improve the signal-to-noise ratio of weak fault-induced impulse responses. This provides valuable analytical insights for the fault diagnosis of rolling bearings in critical escalator components. However, these methodologies are primarily based on signal processing techniques, which have inherent limitations in handling non-stationary and nonlinear signals. Conventional single-domain approaches, such as Fourier transform, are particularly inadequate for capturing time-varying characteristics within transient signals. Furthermore, such unitary signal processing frameworks lack adaptability to diverse operational conditions that induce heterogeneous signal signatures in electromechanical systems. Given that escalator fault signals are intrinsically characterized by nonlinear dynamics and non-stationary behaviors under changing operating conditions, relying on singular signal processing paradigms is insufficient for efficient fault diagnosis.

You et al. [27] proposed a wavelet threshold denoising algorithm that uses Ensemble Empirical Mode Decomposition (EEMD) to process vibration signals from escalator step guides, main drive shafts, and drive mainframes. The extracted fault features were then classified using a Support Vector Machine (SVM) model optimized by Particle Swarm Optimization (PSO–SVM). This method takes advantage of EEMD’s ability to handle non-stationary and nonlinear data due to its self-adjusting decomposition scale characteristics, as well as the wavelet threshold denoising’s superior time–frequency localization properties. However, both EEMD and wavelet threshold denoising are strongly dependent on hyperparameter configurations: the amplitude of injected white noise and ensemble numbers in EEMD have a significant impact on signal decomposition efficacy, whereas the conventional SVM framework is limited to binary classification tasks. Multi-class fault diagnosis necessitates training several SVM classifiers, which adds significant computational overhead and operational inefficiency.

Wang et al. [28] created a fault diagnosis framework for escalator anchor bolt loosening by combining Empirical Wavelet Transform (EWT) with Gray Gradient Co-occurrence Matrix (GGCM) feature extraction and a Bidirectional Long Short-Term Memory (BiLSTM) network. Compared to Empirical Mode Decomposition (EMD) and Variational Mode Decomposition (VMD), EWT achieves faster decomposition rates while reducing mode aliasing artifacts, making it widely applicable in mechanical vibration analysis. The GGCM method effectively extracts fault features from Ensemble Mode Functions (EMFs) while reducing Gaussian noise interference. Nevertheless, this method necessitates expert-dependent hyperparameter tuning, including EWT’s frequency band segmentation parameters, decomposition levels, and GGCM’s grid partitioning thresholds, where suboptimal parameter selection may compromise diagnostic robustness. Accurate fault identification necessitates extensive prior knowledge of signal processing and mechanical system dynamics.

The aforementioned methodologies combine signal processing and deep learning, with high-quality fault features extracted through signal preprocessing before feeding data into neural networks, improving diagnostic performance. These multistage hybrid fault diagnosis approaches effectively combine the advantages of signal processing and deep learning techniques. However, conventional methods, such as wavelet decomposition, Variational Mode Decomposition (VMD), Empirical Wavelet Transform (EWT), and Gray Gradient Co-occurrence Matrix (GGCM), rely heavily on manual parameter tuning, require significant prior knowledge, and have limited model generalization capabilities.

Duan Yibo et al. [29] proposed a CNN–Transformer hybrid network for diagnosing bearing faults in escalators. Tan Botao et al. [30] developed a wavelet-denoising-enhanced CNN–LSTM framework for bearing fault detection, in which wavelet-based noise suppression preserves critical fault signatures while mitigating training interference from signal artifacts. Zheng Yan developed an escalator anchor bolt loosening diagnosis method that uses Empirical Wavelet Transform (EWT) and bispectral analysis to generate multi-scale fault feature vectors, which are then classified using a BiLSTM network. Cao Jingsheng et al. [31] developed an optimized VMD–CNN–BiLSTM architecture for motor bearing fault diagnosis, using an adaptive whale optimization algorithm (AWOA) to autonomously determine the optimal number of Variational Mode Decomposition (VMD) modes and penalty factors, significantly reducing reliance on expert knowledge. Jiang et al. developed [32] a DAE–BiLSTM model for precise fault diagnosis in escalator main drive bearings, in which a deep autoencoder (DAE) performs the dimensional reconstruction of vibration features using unsupervised representation learning, followed by BiLSTM-based temporal pattern recognition to eliminate feature redundancy and improve diagnostic robustness.

However, escalators operate in complex environments with variable working conditions, which typically include significant background noise. Although the aforementioned models can help to reduce noise through signal preprocessing, they often fail to adapt to noise with varying intensities and spectral characteristics across different operational scenarios. Crucially, these methods lack the ability to dynamically adjust their parameters in real time when confronted with strong noise interference in practical conditions. As a result, the diagnostic accuracy and reliability of such approaches are still not fully guaranteed in field applications. Unless the issue of strong noise interference across diverse working conditions is effectively addressed, the advancement of escalator fault diagnosis technologies will be significantly hampered. This limitation not only restricts technical progress, but also complicates equipment maintenance and fault prevention in real-world engineering applications. Wang Zisheng et al. [33] proposed the WPPM-snn model, which is a composite defect detection method applied in variable operating condition fault diagnosis. It is capable of enhancing diagnostic performance under multiple operating conditions by combining the actions of various compound defects.

To address the aforementioned challenges, this study proposes an end-to-end time–frequency dual-domain adaptive diagnostic framework consisting of an improved Residual Convolutional Denoising Autoencoder (RCDAE) and a CNN–Transformer fusion network. The RCDAE achieves the multi-condition adaptive denoising of vibration signals using a Dynamically Multimodal Synergistic (DMS) loss function that combines Mean Squared Error (MSE) and Structural Similarity Index (SSIM), eliminating the need for manual parameter tuning in conventional methods. Furthermore, a hybrid RCDAE–CNN–Transformer architecture is created to extract local transient features using convolutional layers while capturing long-range temporal dependencies with Transformer modules. A cross-attention mechanism is introduced to enable dynamic complementary fusion of time–frequency features, which significantly improves discriminative representation capability for weak fault signatures in the presence of strong noise.

## 2. Common Neural Network Models

### 2.1. CNN Network

Convolutional Neural Networks (CNNs) are a deep learning framework specifically designed for processing grid-like data. Their core principles include local receptive fields, weight sharing, and hierarchical feature extraction. In traditional neural networks, each neuron connects to every pixel in the input layer, resulting in a massive parameter count. In contrast, CNNs significantly reduce the number of required parameters by restricting each neuron to connect only to local regions of the input layer. This design directs the model’s computational focus to the most significant regions of the input data. The most critical function of the convolutional layer is to extract features from the input data. Its convolutional kernel slides sequentially over the input data, extracting specific patterns using dot product operations. Over time, convolutional structures have evolved into various variants, including dilated convolution, which inserts holes between kernel elements to expand the receptive field without increasing the number of parameters, making it suitable for feature extraction from long-term time series data. Additional variants include transposed convolution, depthwise separable convolution, and grouped convolution. Today, convolutional neural networks have advanced significantly, leading to further developed architectures, such as VGGNet [34], ResNet [35], and ResNeXt [36].

### 2.2. Transformer Network

The Transformer [37] is a neural network architecture based solely on the self-attention mechanism. Its core design abandons traditional recurrent and convolutional structures, resulting in natural language processing (NLP) breakthroughs through parallelized training and global dependency modeling. It has since been applied to computer vision, speech recognition, and other fields, demonstrating an exceptional performance on time series data for fault diagnosis. The overall Transformer structure consists of an encoder–decoder framework. The encoder–decoder architecture is commonly used for sequence generation tasks, such as translation, while the encoder-only architecture is appropriate for classification tasks and the decoder-only architecture is designed for generation tasks.

In time series fault diagnosis, the raw data is transformed linearly based on the model’s dimensionality, followed by positional encoding, feature extraction, and classification output to achieve fault diagnosis. The most critical component is the self-attention mechanism, which allows each element in the sequence to interact with all other elements through weighted aggregation to generate new representations. This mechanism addresses the limitations of traditional recurrent networks in capturing long-term dependencies and their inability to support parallel computation. Through extensive innovation by researchers, numerous variants of the self-attention mechanism have emerged, such as sparse attention, multi-head attention, and cross-attention mechanisms.

### 2.3. Autoencoder Network

**Figure 1:**
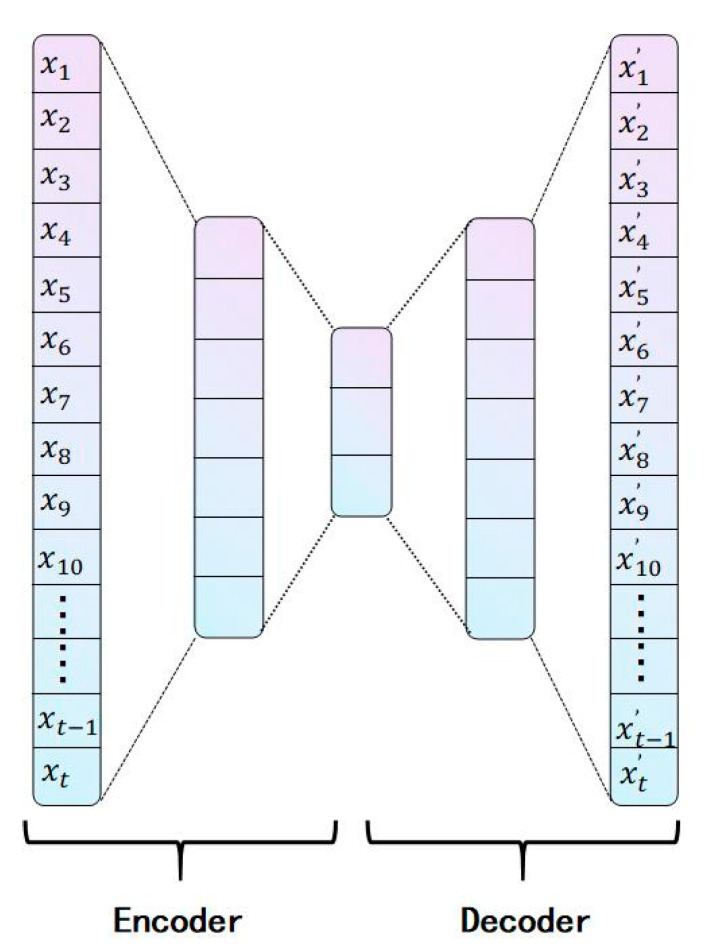
Autoencoder network structure diagram.

An autoencoder [38] is a conventional unsupervised neural network model that consists of the following two key components: an encoder and a decoder. The encoder extracts features from high-dimensional input signals, while the decoder reconstructs the low-dimensional features back into high-dimensional signals. The basic structure of the autoencoder is illustrated in Figure 1. Assuming the original input data is $X=\{x_{1},\;x_{2},\;x_{3},\;x_{4},\;x_{5},\;x_{6},\;x_{7}\ldots \;x_{t-1},\;x_{t}\}$, it first passes through the encoder. In the first fully connected (FC) layer, the n-dimensional data is compressed to seven dimensions. Subsequently, the second FC layer further compresses the seven-dimensional data to three dimensions, obtaining a low-dimensional feature representation; this process constitutes the encoding stage. The three-dimensional data is then expanded back to seven dimensions via an FC layer, followed by another FC layer that reconstructs it into n-dimensional data, yielding the decoder’s output $X^{\prime }=\{{x_{1}}^{\prime },\;{x_{2}}^{\prime },\;{x_{3}}^{\prime },\;{x_{4}}^{\prime },\;{x_{5}}^{\prime },\;{x_{6}}^{\prime },\;{x_{7}}^{\prime }\ldots \;{x_{t-1}}^{\prime },\;{x_{t}}^{\prime }\}$. By comparing the similarity of the input X to the reconstructed output $X^{\prime }$, the reconstruction performance of the autoencoder can be evaluated. The reconstruction performance largely reflects the effectiveness of the autoencoder in condition monitoring.

## 3. Algorithmic Framework

**Figure 2:**
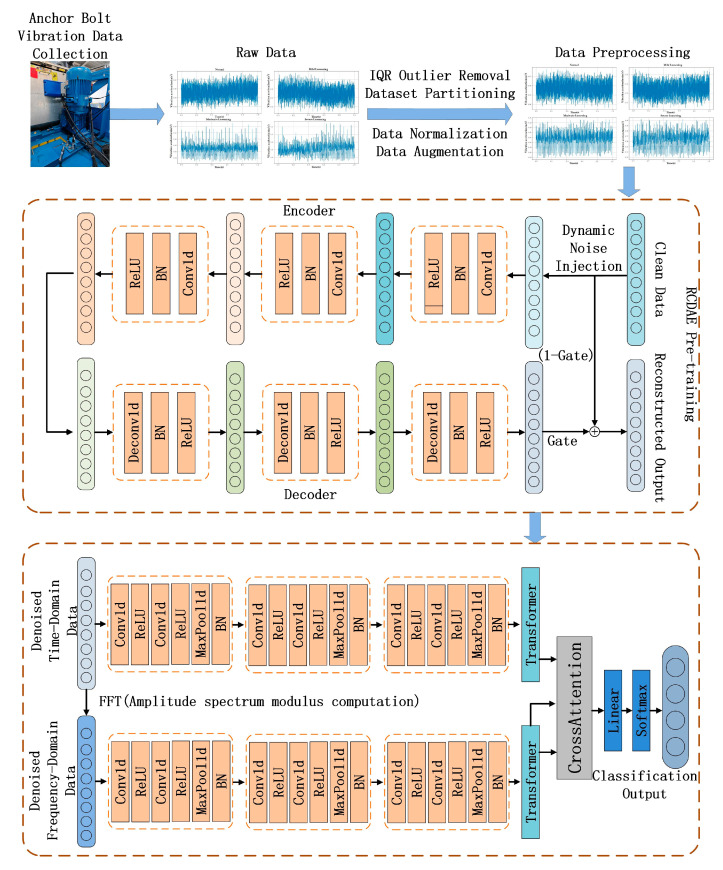
Architecture of the time–frequency RCDAE–CNN–Transformer network.

This section elaborates on the proposed improved RCDAE–CNN–Transformer hybrid network architecture, which is specifically designed for multi-condition anchor bolt loosening fault classification in escalator drive mainframes operating under high-intensity noise environments. As shown in Figure 2, the framework comprises the following three functionally specialized modules: (1) the improved RCDAE denoising module, which implements residual convolutional-based signal purification; (2) the time–frequency hybrid CNN–Transformer network for dual-domain feature extraction; and (3) the cross-attention fusion module, which enables the adaptive integration of complementary time–frequency representations.

### 3.1. Structure of Improved High-Strength Denoising RCDAE Model

#### 3.1.1. DMS Loss Function

Traditional denoising autoencoders use the Mean Squared Error (MSE) [39] as the loss function, which measures the difference between denoised and clean data. However, MSE loss has significant limitations: it only focuses on pixel-level errors by directly quantifying numerical differences, failing to capture the data’s structural characteristics. This constraint makes MSE especially ineffective for processing time series data, as it calculates errors at each timestep while ignoring inherent temporal dependencies within the sequence.

Furthermore, MSE loss has a higher sensitivity to noise due to its error amplification mechanism using squared differences. In time series applications, sensor inaccuracies or environmental interference often cause MSE loss to overestimate noise-related artifacts, reducing denoising performance. The mathematical formula for the MSE loss function is as follows:

(1) $$\mathrm{MSE}=\frac{1}{N}\sum_{i=1}^{n}{\left(y_{i}-{\hat{y}}_{i}\right)}^{2}$$

where N is the sample size; y denotes the target value; and $\hat{y}$ represents the model output.

The Structural Similarity Index (SSIM) [39] loss function is a metric for determining dataset similarity that emphasizes structural coherence over pixel-level differences. SSIM outperforms MSE loss in processing time series data by maintaining temporal coherence.

SSIM loss, which was originally developed for image processing, quantifies similarity between images by comparing their luminance, contrast, and structural information. Owing to its robust structural feature extraction capability, SSIM has demonstrated effective applicability to one-dimensional time-series data analysis. As a result, an increasing number of researchers have used SSIM loss to assess temporal pattern similarity in sequential data. The mathematical formula for the SSIM loss function is as follows:

(2) $$SSIM(x,y)=\frac{\left(2\mu_{x}\mu_{y}+C_{1})(2\sigma_{xy}+C_{2}\right)}{\left(\mu_{x}^{2}+\mu_{y}^{2}+C_{1})(\sigma_{x}^{2}+\sigma_{y}^{2}+C_{2}\right)}$$

where x is the model output; y denotes the target value; $\mu_{x}$ and $\mu_{y}$ represent the mean values of x and y, respectively; $\sigma_{x}^{2}$ and $\sigma_{y}^{2}$ represent the variances of values for x and y, respectively; $\sigma_{xy}$ denotes the covariance between x and y; and $C_{1}$ and $C_{2}$ are small constants for numerical stability.

Traditional MSE loss functions in time series data processing have significant limitations because they overlook structural characteristics in sequential data, resulting in a poor denoising performance. In contrast, the SSIM loss function addresses these shortcomings by quantifying the structural similarity between datasets.

However, because the magnitude scales of the MSE and SSIM losses differ, a direct linear combination is not feasible. Current practices often employ manually fixed weights to balance these losses, but such heuristic tuning requires significant domain expertise. Moreover, the optimal weight ratio varies significantly between operational scenarios, making fixed-weight strategies fundamentally limited.

To address this, we propose a Dynamically Multimodal Synergistic (DMS) loss function that adaptively combines the MSE and SSIM losses to assess the difference between denoised and clean data. However, existing frameworks for adaptive loss functions often exhibit slow convergence and suboptimal weighting of loss components. Traditionally, elements of multi-part loss functions are weighted equally, or their weights are calculated using heuristic methods that produce near-optimal (or suboptimal) results. To address this issue, Rengasamy et al. [40] developed a set of methods known as SoftAdapt, which dynamically adjust the component weights of multi-part loss functions based on the real-time performance statistics of each loss component. SoftAdapt is mathematically intuitive, computationally efficient, and easy to implement. The authors also tested their method on image reconstruction (sparse autoencoders) and synthetic data generation (introspective variational autoencoders), demonstrating its efficacy.

Referring to the above method, the DMS loss function obtained in this paper is as follows:

(3) $$DMS={\mathrm{mse}}_{\mathrm{wight}}\times mse_{loss}+{\mathrm{ssim}}_{\mathrm{wight}}\times ssim_{loss}$$

The dynamic weight adjustment mechanism is formulated as follows:

(4) $${\mathrm{mse}}_{\mathrm{wight}}=\frac{ssim_{loss}}{mse_{loss}}$$

(5) $${\mathrm{ssim}}_{\mathrm{wight}}=\frac{mse_{loss}}{ssim_{loss}}$$

where ${\mathrm{mse}}_{\mathrm{wight}}$ and ${\mathrm{ssim}}_{\mathrm{wight}}$ are dynamic weights and $mse_{loss}$ and $ssim_{loss}$ denote loss values.

During neural network training, the magnitudes of various loss functions can vary significantly, and the loss values themselves evolve as training progresses. Fixed weighting increases the risk of one loss dominating while suppressing the contribution of another. Dynamically adjusting weights ensures that both losses play appropriate roles at different stages of training, thereby optimizing overall model performance.

During model backpropagation, the coefficients ${\mathrm{mse}}_{\mathrm{wight}}$ and ${\mathrm{ssim}}_{\mathrm{wight}}$ act on the gradients. The core of dynamic weighting is to adjust the weights of the gradients from the two loss functions rather than directly modifying the weights of the loss functions themselves. Assume that at any given moment, the MSE loss value is large while the SSIM loss value is small. Here, ${\mathrm{ssim}}_{\mathrm{wight}}$ would receive a smaller weight ${\mathrm{mse}}_{\mathrm{wight}}$, while ${\mathrm{mse}}_{\mathrm{wight}}$ would receive a larger weight, reducing the gradient of MSE loss. Conversely, the gradient of SSIM loss would be amplified. This achieves dynamic equilibrium and prevents the model from overemphasizing either pixel-level similarity or structural similarity.

After the aforementioned processing, the DMS loss function excels at preserving finer input data details during denoising tasks while improving model generalization, making it especially useful in complex denoising scenarios. DMS loss’s dynamic weight adaptation mechanism adapts to data distribution shifts, allowing the RCDAE model to reconstruct data and restore the original data structure to the greatest extent. This dual optimization ensures the optimal retention of discriminative features required for subsequent fault classification while suppressing noise artifacts, resulting in high-quality inputs for downstream diagnostic models.

#### 3.1.2. RCDAE Architecture

**Figure 3:**
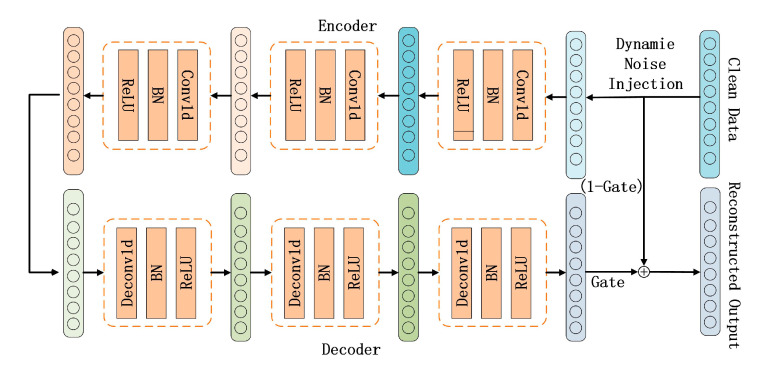
RCDAE network structure diagram.

The Improved High-Strength Denoising RCDAE network consists of symmetrically structured encoders and decoders. The encoder is made up of the following three types of convolutional layers: batch normalization (BN) layers and ReLU activation layers, which are used to extract deep features from input data and reduce dimensionality. The convolutional layers capture local features using sliding window operations, the BN layers stabilize data distribution to accelerate model convergence, and the ReLU activation layers improve model expressivity by introducing nonlinearity, resulting in better data fitting. The decoder comprises the following three groups of layers: transposed convolutional layers, BN layers, and ReLU activation layers, which reconstruct low-dimensional features back to their original dimension in order to achieve denoised data reconstruction. The structure of the RCDAE model is shown in Figure 3.

The vibration characteristics induced by escalator bolt loosening faults often distribute across multiple scales. Using only single-scale convolutional operations fails to capture the multi-scale signatures of vibration signals. Therefore, the RCDAE network incorporates a multi-scale feature extraction strategy. Large convolutional kernels expand the effective receptive field to extract global contextual information, resulting in inter-regional correlations that improve pattern recognition capacity for complex mechanical signatures. These large kernels reduce local noise interference through broad context integration, which improves denoising performance and model robustness. Small convolutional kernels capture localized features with a reduced computational complexity, allowing for the progressive extraction and composition of low-level features. Through the multi-layer stacking of small kernels, the receptive field gradually expands while remaining computationally efficient. In the RCDAE encoder design, the first convolutional layer uses large kernels (specified dimensions) to capture global information, while subsequent layers use small kernels (specified dimensions) to extract deep local features. The decoder replicates this configuration with transposed convolutional layers of the same quantity and kernel size, reconstructing input data from the extracted deep features to achieve the RCDAE model’s denoising functionality. The parameters of each convolutional layer in the RCDAE model are shown in Table 1.

#### 3.1.3. Gated Residual Mechanism

He Kaiming et al. [35] proposed the residual mechanism in 2015, representing a significant milestone in deep learning by effectively addressing the degradation problem in deep neural network training. The gated residual mechanism is a network design strategy that combines residual connections and gating factors (Gate), aiming to improve the model’s data reconstruction and noise removal capabilities by adaptively adjusting the information ratio between the residual and identity mapping paths.

The gating mechanism increases the flexibility of residual connections, allowing for adaptive adjustment of the information ratio based on the characteristics of the residual and identity mapping paths. The gating factor is a learnable parameter produced by a gating unit, such as the Sigmoid or Softmax activation functions. The gating factor ranges from zero to one. When it approaches zero, the information from the identity mapping path takes precedence, and the model tends to directly transmit the output data; when the gating factor approaches one, the model output primarily follows the residual path, emphasizing the learning of residual features in the input data. The gated residual mechanism is mathematically formulated as follows:

(6) y=Gate×F(x)+(1−Gate)×x

where Gate is the gating factor; F(x) represents the residual path output; x denotes the input data; and y is the final model output.

Compared to traditional residual connections, the gated residual mechanism is more flexible. The gating factor allows the network to dynamically adjust the information ratio between pathways, rather than relying on fixed parametric residual connections. This adaptive capability provides additional advantages for learning complex patterns in mechanical fault diagnosis.

### 3.2. CNN–Transformer Hybrid Architecture with Time–Frequency Cross-Attention

Vibration signals from escalator drive mainframe anchor bolts are nonlinear, non-stationary complex time series data. While temporal representations reveal time-evolving characteristics, they do not expose frequency-domain signatures. These signals typically contain multiple harmonic components across different frequency bands, resulting in complex waveform patterns in the time domain that resist direct interpretation. Exclusive reliance on temporal analysis ignores global spectral features. Fast Fourier Transform (FFT) efficiently converts signals to frequency-domain representations, emphasizing harmonic amplitudes and spectral distributions, but sacrifices time-varying feature resolution. Consequently, conventional single-domain approaches fail to capture dynamic behavioral patterns, limiting the accuracy of fault diagnosis in the early stages. Integrating time–frequency dual-domain analysis allows for a comprehensive characterization of transient dynamics and inherent vibrational modes, which improves diagnostic precision, robustness, and adaptability.

A dual-channel time–frequency diagnostic framework is designed to process temporal and spectral signals in parallel, resulting in complementary representations of non-stationary nonlinear signatures. The FFT algorithm, an optimized implementation of Discrete Fourier Transform (DFT) [41], efficiently converts discrete temporal data into frequency-domain representations. The mathematical formula for DFT is expressed as follows:

For a discrete signal X[n] of length N, its DFT is defined as follows:

(7) $$X[k]=\sum_{n=0}^{N-1}x[n]\cdot e^{-j\frac{2\pi }{N}kn},\;k=0,\;1,\;\ldots \ldots ,\;N-1$$

where X[k] represents the complex representation of the k-th frequency component in the frequency domain; j is the imaginary unit; and $e^{-j\frac{2\pi }{N}kn}$ denotes the twiddle factor.

Direct computation of Discrete Fourier Transform (DFT) requires $O(N^{2})$ complex multiplications and additions, leading to prohibitive computational costs for large datasets. The Fast Fourier Transform (FFT) algorithm addresses this limitation by employing a divide-and-conquer strategy, which involves recursively decomposing DFT into smaller subproblems, reducing computational complexity to O(NlogN) and significantly improving operational efficiency. In mechanical vibration analysis, FFT has become critical for processing time-domain signals with complex waveforms that obscure fault signatures. By converting these signals to frequency-domain representations, FFT allows for the precise localization of fault-related characteristic frequencies. This capability is especially important in high-noise environments, where the effective separation of noise artifacts from diagnostically relevant signal components is essential.

After FFT, the time-domain data is transformed into a frequency-domain representation of the original signal as a complex sequence. This sequence captures both the amplitude and phase information of different frequency components within the time-domain signal. Since operations such as Conv1d, ReLU, and MaxPool1d cannot directly process complex-valued data, frequency-domain data must first be preprocessed. The magnitude spectrum, obtained by computing the modulus of the complex FFT output, is the optimal representation for frequency-domain data in fault diagnosis. It directly reflects the vibration energy intensity of each frequency component, and different faults appear as abnormal peaks in specific frequency bands, resulting in the most intuitive fault features.

The multi-head attention mechanism in the Transformer projects input data into distinct subspaces via multiple parallel linear transformations, resulting in independent attention heads. This architectural design eliminates inter-head dependencies, allowing for full parallelism during computation. By avoiding sequential processing constraints, the model accelerates the handling of large amounts of time series data while maintaining diagnostic accuracy. The mathematical formula for calculating self-attention weights is as follows:

(8) $$\mathrm{Attention}(Q,K,V)=Soft\max(\frac{Q\cdot K^{T}}{\sqrt{d_{k}}})\cdot V$$

where Q, K, and V stand for query, key, and value vectors, respectively, all linearly projected from the input sequence; $\sqrt{d_{k}}$ denotes the dimensionality of key vectors.

The cross-attention mechanism [42] is an improved variant of the self-attention mechanism in Transformer models. In self-attention, the query (Q), key (K), and value (V) are all derived from the same input, whereas in cross-attention, Q is sourced from one input and K and V from another, allowing for cross-modal feature fusion. This mechanism takes full advantage of the complementary nature of time-domain and frequency-domain features in vibration signals: time-domain features reflect the signal’s temporal variations, whereas frequency-domain features describe its spectral distribution across frequency components. By adaptively fusing these dual-domain representations via cross-attention, the model simultaneously captures diagnostically significant information from both dimensions, increasing the expressive power of fault-related signatures in complex vibration signals.

### 3.3. Time–Frequency RCDAE–CNN–Transformer Model for Escalator Drive Mainframe Anchor Bolt Loosening Diagnosis

In conclusion, the workflow of the proposed time–frequency RCDAE–CNN–Transformer network model is as follows:

Step 1: RCDAE Pre-training: Unsupervised pre-training of the RCDAE model with only the training and validation sets is conducted. During training, Gaussian white noise with intensities ranging from 10 dB to −10 dB is dynamically injected into the training data, while the validation set is always augmented with −10 dB noise. The most optimal model parameters are saved based on the validation results.

Step 2: RCDAE Integration: The pre-trained RCDAE parameters are loaded into the RCDAE–CNN–Transformer framework. The RCDAE module denoises signals to produce high-fidelity, clean data.

Step 3: Frequency-Domain Conversion: Fast Fourier Transform (FFT) is applied to the denoised signals to obtain spectral representations.

Step 4: Dual-Domain Feature Extraction: To extract multi-scale local features, the time-domain signals and frequency-domain spectra are processed separately through CNN layers.

Step 5: Global Dependency Modeling: CNN-derived local features are input into Transformer encoders to capture long-range temporal dependencies across both domains.

Step 6: Cross-Attention Fusion: Frequency-domain features are used as key and value vectors, while time-domain features serve as query vectors. Cross-attention is implemented for adaptive time–frequency feature fusion.

Step 7: Fault Classification: Diagnosis is finalized through a fully connected layer using SoftMax activation, which produces probabilistic fault category predictions.

## 4. Experimental Validation

### 4.1. Test Platform

The experimental platform used a custom-configured computer system running 64-bit Windows 10 with an Intel^®^ Core™ i7-9750H CPU @ 2.60 GHz. The neural network architecture was implemented using the PyTorch 2.4.1 framework.

### 4.2. Data Acquisition Protocol

#### 4.2.1. Raw Data Acquisition

**Figure 4:**
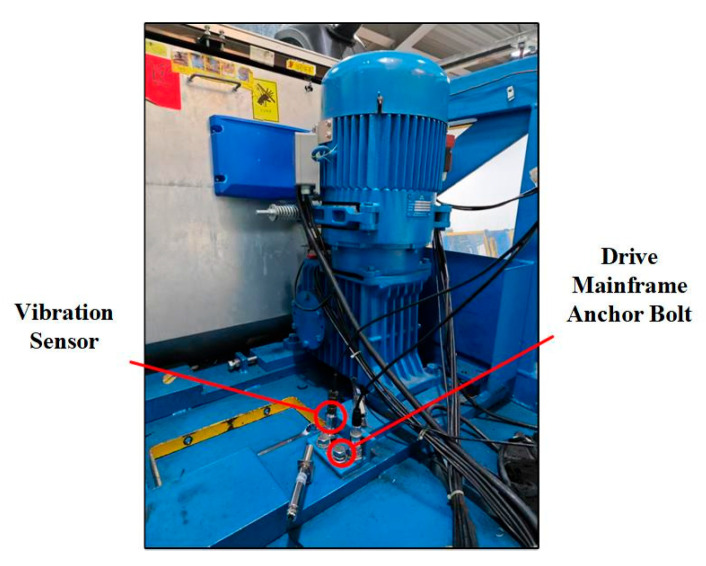
Test platform.

**Figure 5:**
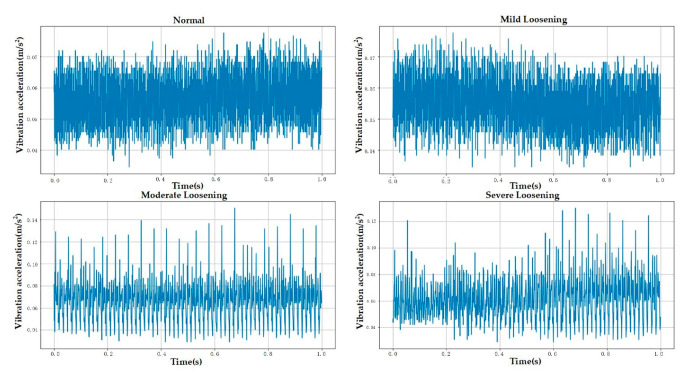
Raw data.

**Figure 6:**
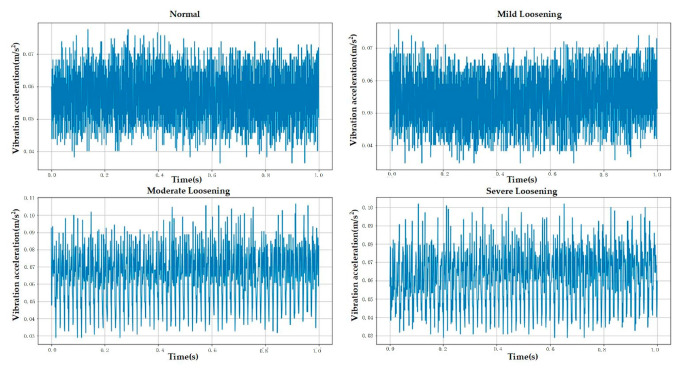
Sanitized data (post-outlier removal).

The experimental data used in this study were collected from an Otis escalator with a rated operating speed of 0.5 m/s and an inspection speed of 0.3 m/s. All experiments were conducted while the escalator operated in inspection mode. The sensor employed was a piezoelectric single-axis vibration sensor with a sampling frequency of 1000 Hz, recording each fault category for 5 min. The sensor was mounted at the location shown in Figure 4 using a magnetic base. The experiment collected data for anchor bolts in four different loosening states. The escalator bolts were of the M16 specification and tightened to a nominal torque of 130 Nm. Consequently, mild, moderate, and severe loosening were defined as torque values equivalent to 90%, 80%, and 70% of the tightening torque, respectively [28,43]. Raw data contained outliers caused by abnormal mechanical impacts during acquisition, which could compromise diagnostic accuracy. This study used the Interquartile Range (IQR) method to detect and eliminate these anomalies. Given the small number of outliers (less than 0.5% of total samples), removing them had no significant effect on the underlying data distribution. Figure 4, Figure 5 and Figure 6 demonstrate the experimental setup, representative raw signals, and sanitized datasets.

The sanitized vibration data was divided into samples with 1024 data points each. To expand the dataset, a sliding window data augmentation method with a 0.5 overlap ratio was used, and it was then partitioned into training, validation, and test sets in the ratio 0.7:0.2:0.1. 

Table 2 details the distribution of fault categories.

#### 4.2.2. Noisy Data Simulation

To validate the model performance in high-intensity noise environments, Gaussian white noise is injected into pristine data to simulate extreme noise conditions. This approach is justified because both sensor thermal noise and multi-source hybrid background noise in industrial settings approximate Gaussian distributions. The most physically representative simulation method is to introduce white Gaussian noise of varying intensities, with noise intensity levels quantified using the Signal-to-Noise Ratio (SNR) [44], defined as follows:

(9) $${\mathrm{SNR}}_{db}=10\;\log_{10}(\frac{P_{signal}}{P_{noise}})$$

where $P_{\mathrm{signal}}=\frac{1}{N}{\sum_{i=1}^{N}\left|s_{i}\right|}^{2}$, $P_{\mathrm{noise}}=\frac{1}{N}{\sum_{i=1}^{N}\left|n_{i}\right|}^{2}$, N denotes the number of sample points, $s_{i}$ represents the discrete pristine vibration signal acquired by the sensor at the i-th time point, and $n_{i}$ is the instantaneous value of Gaussian white noise superimposed on the clean signal at the same temporal position.

**Figure 7:**
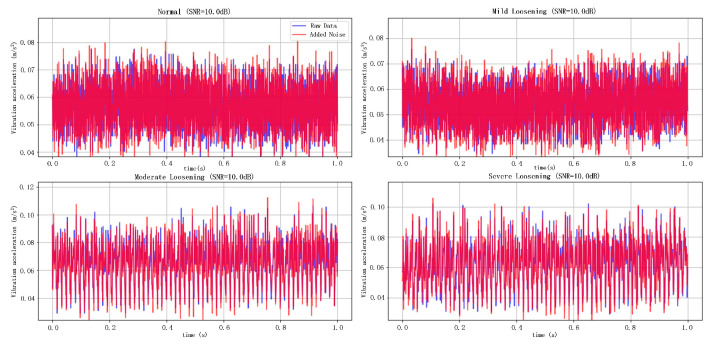
Simulation of 10 dB noisy data.

**Figure 8:**
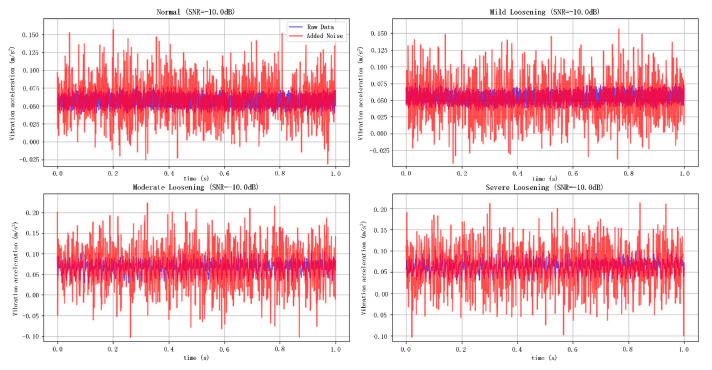
Simulation of −10 dB noisy data.

As shown in Figure 7 and Figure 8, the noise-contaminated data illustrates the original signal and its counterparts contaminated with Gaussian white noise at SNR levels of 10 dB and −10 dB, respectively. The blue curves represent the pristine signals, while the red curves depict the noise-injected signals. Visual analysis reveals significantly greater corruption under the −10 dB noise scenario than in the 10 dB case, demonstrating the efficacy of variable-intensity Gaussian noise injection for emulating high-intensity background interference in operational environments.

### 4.3. Model Training and Performance Visualization

#### 4.3.1. Model Training

The hyperparameters configured during model training were as follows: The RCDAE model was trained for a maximum of 200 epochs, while the RCDAE–CNN–Transformer network went through 40 epochs. Both models used an initial learning rate of 0.0003 and a dynamic scheduler that reduced the rate every ten epochs, with a batch size of 32. The RCDAE used the Adamw optimizer with the DMS loss function, while the RCDAE–CNN–Transformer used the Adam optimizer with cross-entropy loss. The aforementioned hyperparameters were all determined using expert insights and multiple iterative experiments.

**Figure 9:**
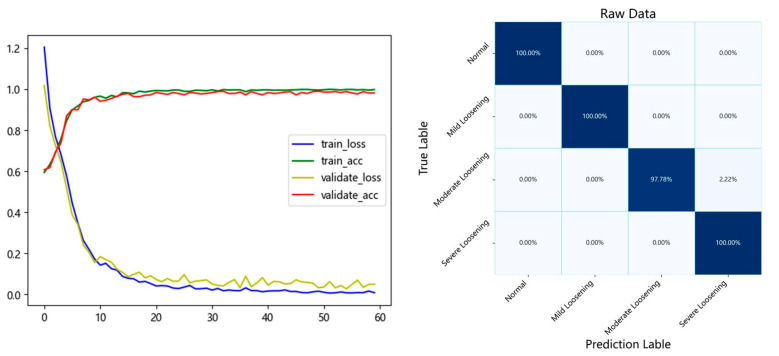
Training convergence and test set confusion matrix.

The model was trained using the field-acquired data and hyperparameters listed above, and the trained parameters were saved once completed. The test set was evaluated for performance, and the average classification accuracy across multiple experimental trials was 99.55%. The training convergence curves and test set confusion matrix are presented in Figure 9.

#### 4.3.2. Performance Comparison of Different Models on Raw Data Set of Bolt Loosening

The proposed model outperforms the others. Table 3 presents a comparison of the performance metrics of our model and other models. Among the three models using CNN–Transformer as the main architecture, the CAE–CNN–Transformer model fails to achieve efficient denoising. On the contrary, it leads to a lower diagnostic performance than the CNN–Transformer framework because it loses a lot of effective information during feature compression. The experimental results show that the proposed model achieves a 99.56% accuracy on the original noise-free data, which is better than all comparison models in multiple evaluation indicators.

#### 4.3.3. Performance Comparison of Different Models on Bolt Loosening Noise Dataset

Given that most mechanical equipment operates in environments with significant noise interference, this study conducted experiments using data contaminated with varying levels of Gaussian white noise. To simulate this, noise was introduced by adding Gaussian white noise to the original clean data, with noise intensity measured using the Signal-to-Noise Ratio (SNR). The proposed model achieved an average test set accuracy of 93.88% across noise levels ranging from 10 dB to −10 dB. Table 4 shows the test set accuracy across different models for noise levels ranging from 10 dB to −10 dB.

It is observable that diagnostic accuracy across all models declines as noise intensity increases, which is consistent with real-world operational patterns. In contrast to comparative models, the proposed architecture exhibits a gradual degradation in accuracy under varying noise levels, demonstrating a superior adaptability to multi-intensity noise profiles. This characteristic renders it particularly suitable for escalator monitoring scenarios characterized by complex environmental interference and variable load conditions. Among benchmark models, 1DCNN has the lowest performance, a result of its simplistic sequential convolutional structure, which inherently compromises noise immunity.

#### 4.3.4. Validation Experiment of DMS Loss Function on Bolt Loosening Dataset

To validate the effectiveness of the proposed DMS loss function, comparative experiments with various loss functions were conducted on the same model architecture. The experiments differed only in the loss functions used, as follows: (1) MSE loss alone, (2) SSIM loss alone, (3) a fixed-weight combination of MSE and SSIM losses (weight coefficients of 0.4 and 0.6), and (4) the proposed DMS loss. Table 5 shows that the DMS loss function group consistently outperformed the three benchmark groups in terms of test accuracy across a variety of noise-contaminated datasets. Notably, the fixed-weight group underperformed even compared to the single-loss groups, due to its inability to reconcile the magnitude discrepancy between constituent loss functions. The static weighting strategy failed to synergistically combine their advantages while, paradoxically, reducing the individual loss functions’ efficacy. This comparative experiment empirically validates the effectiveness of the proposed DMS loss function.

### 4.4. CWRU Bearing Dataset Experiments

#### 4.4.1. Performance Comparison of Different Models on Raw Data Set of CWRU Bearing Dataset

To validate the performance of the proposed model in other scenarios, experiments were conducted using the CWRU bearing dataset under identical experimental conditions. Table 6 shows the comparative results of different models on the original dataset without any added noise. Model performance was assessed using the following four metrics: Precision, Recall, F1-score, and Accuracy. The experimental results demonstrated that the proposed model significantly outperformed all comparative models across all four evaluation metrics.

#### 4.4.2. Performance Comparison of Different Models on CWRU Bearing Noise Dataset

The accuracy rates of different models across datasets with varying levels of noise are presented in Table 7. Notably, the proposed model achieves an average accuracy of 94.44% across noise-contaminated datasets. In contrast, 1DCNN and WDCNN produce suboptimal diagnostic results due to their simplistic architectures and consequently weaker noise immunity, while the proposed model consistently outperforms all comparative models in performance under each noise condition.

#### 4.4.3. Validation Experiment of DMS Loss Function on CWRU Bearing Dataset

To validate the effectiveness of the proposed DMS loss function, comparative experiments were conducted across datasets with varying noise intensities using identical loss function configurations, as described previously. The results presented in Table 8 convincingly demonstrate the robustness and efficacy of the DMS loss function under a variety of noise conditions.

## 5. Results and Discussion

### 5.1. Signal Contribution Weight Visualization

**Figure 10:**
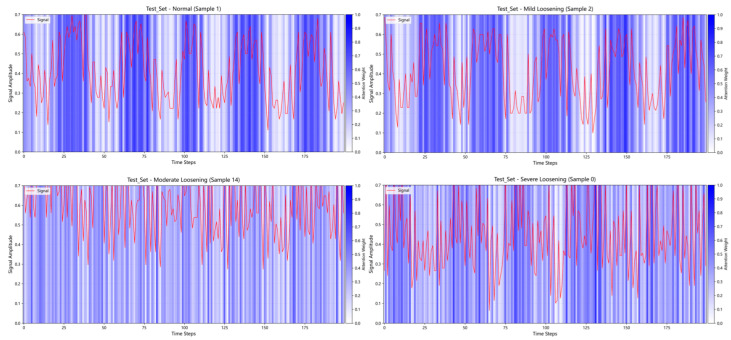
Visualization of signal contributions for samples under different conditions.

To demonstrate that the RCDAE model assigns varying weights to different segments of the raw signal, Figure 10 depicts the signal contribution weights of denoised signals after processing by the RCDAE model. A subset of 200 data points is extracted for this visualization.

Figure 10 depicts the signal contributions for one sample from each of the four conditions in the test set. The red curves depict segments of the original vibration signals, while the blue bars represent the contribution levels of the signals to the RCDAE model. Darker blue colors represent a greater contribution of those signal segments to the RCDAE model. The RCDAE model focuses primarily on these darker-colored signal regions, assigning them larger weights. Further investigation reveals that the RCDAE model focuses primarily on peak regions within the raw signals. This occurs because vibrational impacts on bolts typically concentrate the most critical information within these peak segments. By assigning different weights to different signal segments, the RCDAE model effectively increases the proportion of valuable information in the raw signals, thereby improving diagnostic performance in downstream tasks. The figure indicates that the maximum weight values approach 1.0 while the minimum weights approach 0.0, demonstrating that different temporal data points contribute differently to the denoising process of the RCDAE model. This weighting mechanism allows the model to amplify essential features through higher weights while suppressing redundant information via lower weights, significantly improving its denoising capability.

### 5.2. Visual Chart for Analysis of Bolt Loosening Experimental Results

**Figure 11:**
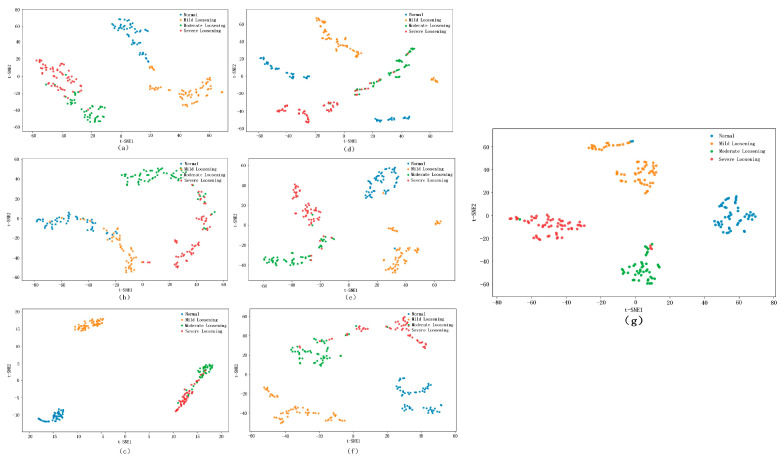
The visualization of the features of the fully connected layer using t-SNE. (**a**) 1DCNN, (**b**) WDCNN, (**c**) MA1DCNN, (**d**) CNN–BiLSTM, (**e**) CNN–Transformer, (**f**) CAE–CNN–Transformer, and (**g**) RCDAE–CNN–Transformer.

In the t-SNE visualization of features extracted from the fully connected layer, where points of different colors represent samples from different states, superior models exhibit tighter clustering of same-color points and clearer separation between differently colored clusters. The fully connected layer feature t-SNE visualization of the bolt loosening fault dataset is shown in Figure 11, from which it can be seen that the proposed model demonstrates a superior classification efficacy on the test set, as evidenced by its compact intra-cluster distribution and well-separated inter-cluster boundaries. Compared to other models, it has significant advantages in both intra-cluster cohesion and inter-cluster distinctiveness.

The Silhouette Coefficient [47] is a metric for determining the quality of clustering results. It quantifies the validity of clustering by measuring both the cohesion of samples within their own clusters and their separation from other clusters. This coefficient represents both intra-cluster compactness and inter-cluster separation. Intra-cluster compactness reflects the similarity between a sample and its assigned cluster, with lower values indicating that samples are closer to the cluster center and clusters are more tightly grouped. Inter-cluster separation reflects the degree of distinction between a sample and other clusters, with higher values indicating a greater distance from other clusters and clearer boundaries between clusters.

The Silhouette Coefficient for sample i is defined as follows:

(10) $$s(i)=\frac{b(i)-a(i)}{\max(a(i),\;b(i))}$$

For sample i, a(i) represents the intra-cluster average distance of the sample, while b(i) denotes the average distance from sample i to the nearest neighboring cluster.

The overall Silhouette Coefficient for the entire dataset is the mean value of s(i) across all samples, as follows:

(11) $$S(N)=\frac{1}{N}\sum_{i=1}^{N}s(i)$$

where N denotes the sample size and S(N) represents the global Silhouette Coefficient of the entire dataset. A value closer to one indicates a better clustering quality. As shown in Table 9, the proposed model achieves a Silhouette Coefficient closer to one than other models, confirming its superior classification performance across all comparative models.

### 5.3. Visual Chart for Analysis of CWRU Bearing Dataset Experimental Results

**Figure 12:**
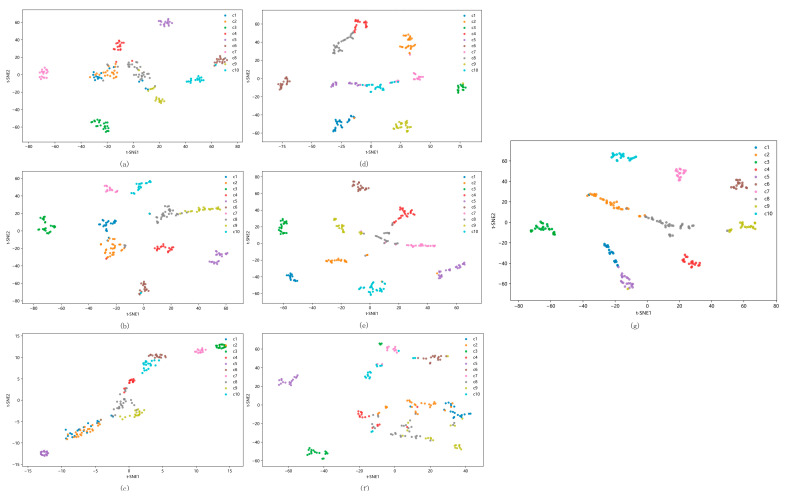
The visualization of the features of the fully connected layer using t-SNE on CWRU. (**a**) 1DCNN, (**b**) WDCNN, (**c**) MA1DCNN, (**d**) CNN–BiLSTM, (**e**) CNN–Transformer, (**f**) CAE–CNN–Transformer, and (**g**) RCDAE–CNN–Transformer.

As shown in Figure 12, the proposed model exhibits the tightest intra-cluster compactness and the most distinct inter-cluster separation, validating its superior feature discriminability. Comparative models demonstrate partial sample separation in t-SNE visualizations but exhibit overlapping clusters for perceptually similar samples, resulting in misclassification during fault diagnosis.

The specific fault location and severity corresponding to different categories in the above t-SNE diagram are shown in Table 10.

Table 11 shows the Silhouette Coefficients for the CWRU bearing fault dataset across different models. The proposed model achieves values closer to one, indicating a superior classification performance.

## 6. Conclusions

To address the issue of weak vibration signals being susceptible to strong noise interference in the early-stage loosening fault diagnosis of escalator drive mainframe anchor bolts, this study proposes a time–frequency RCDAE–CNN–Transformer model with dual-domain feature fusion. The model improves noise immunity by dynamically pre-training a Residual Convolutional Denoising Autoencoder (RCDAE), combines local time-domain features extracted by CNN with global frequency-domain dependencies captured by Transformer, and addresses feature deficiencies in traditional single-domain analysis through the cross-attention-mechanism-based fusion of complementary time–frequency information. The experimental results show that the proposed model achieves an average diagnostic accuracy of 93.88% across noise intensities ranging from 10 dB to −10 dB. In cross-domain validation using the CWRU bearing dataset, it achieves a 94.44% accuracy in noisy environments, indicating strong generalizability and engineering applicability. However, the current study is based on fixed sensor configurations. Future research will optimize sensor placement strategies, investigate coordinated diagnostic methods for simultaneous loosening localization and severity quantification to improve fault localization precision, integrate multi-source sensor fusion technology to enhance diagnostic performance, and deploy the model on embedded devices for field trials.

## Figures and Tables

**Table 1:** Parameters of each convolutional layer in the RCDAE network.

Encoder	Conv Layer Parameters (In, Out, Kernel Size, Stride, Padding)
Conv1d_1	(1, 64, 11, 0, 5)
Conv1d_2	(64, 128, 3, 2, 1)
Conv1d_3	(128, 256, 3, 2, 1)
Decoder	Conv layer parameters (in, out, kernel size, stride, padding)
Conv1d_1	(256, 128, 3, 2, 1)
Conv1d_2	(128, 64, 3, 2, 1)
Conv1d_3	(64, 1, 11, 0, 5)

**Table 2:** Fault category specifications.

Class	Fault Location	Severity Level	Sample Count
0	Drive Mainframe Anchor Bolt	Normal	580
1	Drive Mainframe Anchor Bolt	Mild Loosening	580
2	Drive Mainframe Anchor Bolt	Moderate Loosening	580
4	Drive Mainframe Anchor Bolt	Severe Loosening	580

**Table 3:** Performance comparison of different models on raw data set of bolt loosening.

Model	Precision (%)	Recall (%)	F1-Score (%)	Accuracy (%)
1DCNN	96.88	96.58	96.58	96.58
WDCNN [45]	95.83	95.65	96.08	95.49
MA1DCNN [46]	93.75	93.57	93.72	93.63
CNN–BiLSTM	98.67	98.66	98.66	98.66
CNN–Transformer	99.17	99.14	99.14	99.14
CAE–CNN–Transformer	94.79	94.52	95.23	94.71
RCDAE–CNN–Transformer	99.55	99.55	99.55	99.55

**Table 4:** Performance comparison of different models on bolt loosening noise dataset.

SNR	10 db	8 db	6 db	4 db	2 db	0 db	−2 db	−4 db	−6 db	−8 db	−10 db
Model	Accuracy (%)										
1DCNN	92.71	83.85	81.25	71.35	61.46	68.23	88.54	65.10	84.90	82.81	79.17
WDCNN	94.27	90.62	91.15	93.23	77.08	78.65	85.42	73.44	89.06	89.58	88.54
MA1DCNN	93.23	84.90	93.75	72.92	66.67	72.40	90.62	70.31	86.46	82.81	85.42
CNN– Transformer	97.32	95.09	95.98	93.75	87.10	87.95	90.62	82.59	91.50	92.41	89.29
CAE–CNN– Transformer	94.79	92.19	92.71	88.54	70.31	79.17	68.75	76.04	89.58	84.38	83.85
RCDAE–CNN– Transformer	98.21	97.77	97.92	94.64	89.73	89.29	94.60	88.40	95.09	93.75	93.30

**Table 5:** Comparative experimental results of different loss functions on bolt loosening datasets.

SNR (db)	No Noise	10 db	8 db	6 db	4 db	2 db	0 db	−2 db	−4 db	−6 db	−8 db	−10 db
MSE	99.48	97.40	94.27	94.79	94.27	88.75	89.01	93.23	86.98	92.19	93.23	92.71
SSIM	99.48	96.88	93.75	95.83	92.19	87.54	85.94	94.27	83.33	93.23	91.25	90.95
Fixed weight	97.92	92.71	84.38	72.40	86.46	66.67	72.40	79.17	78.12	71.88	88.02	81.25
DMS	99.56	98.21	97.77	97.92	94.64	89.73	89.29	94.60	88.40	95.09	93.75	93.30

**Table 6:** Performance comparison of different models on raw data set of CWRU bearing dataset.

Model	Precision (%)	Recall (%)	F1-Score (%)	Accuracy (%)
1DCNN	90.62	90.76	91.42	90.87
WDCNN	93.23	93.79	93.49	93.15
MA1DCNN	88.02	88.99	89.13	88.79
CNN–BiLSTM	95.54	95.88	95.91	95.63
CNN–Transformer	97.32	96.99	97.05	96.95
CAE–CNN–Transformer	94.79	94.86	94.44	94.58
RCDAE–CNN–Transformer	99.11	99.25	99.09	99.15

**Table 7:** Performance comparison of different models on CWRU bearing noise dataset.

SNR (db)	10 db	8 db	6 db	4 db	2 db	0 db	−2 db	−4 db	−6 db	−8 db	−10 db
Model	Accuracy (%)										
1DCNN	79.17	81.77	84.90	84.88	73.44	82.81	89.06	74.48	78.65	79.69	69.27
WDCNN	86.46	86.46	90.62	72.40	85.42	84.90	95.83	81.25	86.46	91.15	82.29
MA1DCNN	83.33	85.42	88.02	75.52	81.77	90.10	95.83	84.38	90.10	90.62	84.38
CNN– Transformer	95.09	96.43	95.09	88.39	92.41	92.86	94.20	91.96	92.41	92.41	93.30
CAE–CNN– Transformer	92.18	94.58	90.17	85.45	88.79	84.56	91.17	85.28	89.87	85.45	90.78
RCDAE–CNN– Transformer	95.54	96.88	96.88	90.62	95.09	93.75	95.54	93.30	94.64	92.89	93.75

**Table 8:** Comparative experimental results of different loss functions on CWRU bearing dataset.

SNR (db)	No Noise	10 db	8 db	6 db	4 db	2 db	0 db	−2 db	−4 db	−6 db	−8 db	−10 db
MSE	97.40	90.62	96.88	94.27	81.77	80.73	93.23	95.48	81.77	87.50	89.06	92.19
SSIM	96.54	95.12	96.35	94.55	83.85	87.50	92.85	94.23	92.19	94.35	91.54	89.58
Fixed weight	96.88	94.79	95.45	96.35	83.33	86.98	93.23	92.25	92.71	93.69	91.77	93.75
DMS	97.77	95.54	96.88	96.88	90.62	95.09	93.75	95.54	93.30	94.64	92.89	93.75

**Table 9:** Silhouette Coefficients of different models on datasets with varying noise intensities.

	1DCNN	WDCNN	MA1DCNN	CNN– Transformer	CAE–CNN– Transformer	RCDAE–CNN– Transformer
No noise	−0.0636	−0.0598	−0.0581	−0.0625	−0.0677	−0.0579
10 db	−0.0498	−0.0550	−0.0691	−0.0575	−0.0487	−0.0472
8 db	−0.0526	−0.0466	−0.1021	−0.0471	−0.0531	−0.0466
6 db	−0.0460	−0.0467	−0.0917	−0.0552	−0.0633	−0.0401
4 db	−0.0321	−0.0477	−0.0661	−0.0669	−0.0552	−0.0411
2 db	−0.0575	−0.0606	−0.0652	−0.0584	−0.0564	−0.0495
0 db	−0.0626	−0.0431	−0.0782	−0.0598	−0.0550	−0.0537
−2 db	−0.0478	−0.0506	−0.0543	−0.0699	−0.0494	−0.0472
−4 db	−0.0673	−0.0606	−0.0697	−0.0621	−0.0522	−0.0506
−6 db	−0.0703	−0.0500	−0.0923	−0.0516	−0.0503	−0.0457
−8 db	−0.0570	−0.0695	−0.0621	−0.0578	−0.0595	−0.0499
−10 db	−0.0678	−0.0737	−0.0896	−0.0694	−0.0629	−0.0601

**Table 10:** Corresponding table of fault types for CWRU bearing data set.

Class	Fault Location	Diameter of Fault
c1	Normal	Normal
c2	Inner	0.007 inch
c3	Ball	0.007 inch
c4	Outer	0.007 inch
c5	Inner	0.014 inch
c6	Ball	0.014 inch
c7	Outer	0.014 inch
c8	Inner	0.021 inch
c9	Ball	0.021 inch
c10	Outer	0.021 inch

**Table 11:** Silhouette Coefficients of different models on CWRU bearing dataset with varying noise intensities.

	1DCNN	WDCNN	MA1DCNN	CNN– Transformer	CAE–CNN– Transformer	RCDAE–CNN– Transformer
No noise	−0.1897	−0.1750	−0.2435	−0.1485	−0.1749	−0.1596
10 db	−0.1980	−0.1591	−0.2273	−0.1551	−0.1511	−0.1367
8 db	−0.1460	−0.1658	−0.1617	−0.1489	−0.1575	−0.1320
6 db	−0.2098	−0.1613	−0.1726	−0.1447	−0.1657	−0.1578
4 db	−0.2073	−0.1462	−0.1504	−0.1452	−0.1541	−0.1293
2 db	−0.1968	−0.1558	−0.2165	−0.1611	−0.1933	−0.1402
0 db	−0.1528	−0.1411	−0.1887	−0.1599	−0.1702	−0.1331
−2 db	−0.1308	−0.1166	−0.1561	−0.1210	−0.1649	−0.1072
−4 db	−0.1691	−0.1512	−0.1692	−0.1544	−0.1801	−0.1390
−6 db	−0.1824	−0.1537	−0.1634	−0.1692	−0.1646	−0.1495
−8 db	−0.1571	−0.1512	−0.1428	−0.1415	−0.1454	−0.1356
−10 db	−0.1578	−0.1578	−0.1494	−0.1618	−0.2066	−0.1314

## Data Availability

The original contributions presented in this study are included in the article. Further inquiries can be directed to the corresponding authors.

## References

[1] Bai J., Zhang D., Sun Y., Tang X., Chen Z. (2024). Cloud-edge Collaboration Oriented Intelligent Edge Diagnosis for Escalator Bearing. Mech. Electr. Eng. Technol..

[2] Li H., Wang Y., Xing Y., Zhao X., Wang K. (2021). Contributing Factors Affecting the Severity of Metro Escalator Injuries in the Guangzhou Metro, China. Int. J. Environ. Res. Public Health.

[3] Saeed A.B., Gitaffa S.A., Dawai R.I. (2025). FPGA-Based Realization of Intelligent Escalator Controller Using Artificial Neural Network. J. Electr. Comput. Eng..

[4] Wu Z., Liu J., Yu F. (2022). Progress of Escalator Online Monitoring and Fault Diagnosis Technology. China Spec. Equip. Saf..

[5] Ma H., Li Z., Wang Z., Feng R., Li G., Xu J. (2021). Research on Measuring Device and Quantifiable Risk Assessment Method Based on Fmea of Escalator Brake. Adv. Mech. Eng..

[6] Wang D. (2022). Research on Escalator Fault Diagnosis Based on Expert Knowledge and Vibration Signal Analysis. Master’s Thesis.

[7] Kahali D., Rastogi R. (2021). Comparative Analysis of Escalator Capacity at Metro Stations: Theory Versus Practice. Transportation.

[8] Li N., Cao Z., Bao W., Lin S., Zou T., Yan M. (2024). Experimental Study and Finite Element Analysis of Heavy-Duty Escalator Truss under Full Load Conditions. Sci. Rep..

[9] Li Z., Zhang X., Wu T., Zhu L., Qin J., Yang X. (2021). Effects of Slope and Speed of Escalator on the Dispersion of Cough-Generated Droplets from a Passenger. Phys. Fluids.

[10] Jiao Z., Lei H., Zong H., Cai Y., Zhong Z. (2022). Potential Escalator-Related Injury Identification and Prevention Based on Multi-Module Integrated System for Public Health. Mach. Vis. Appl..

[11] Zhang Z., Tong Y., Jiang X., Yang L. Requirements Analysis and Overall Design of Escalator Condition Monitoring and Early Warning System. Proceedings of the Seventh International Conference on Mechatronics and Intelligent Robotics (ICMIR 2023).

[12] Tang X. (2023). Research on Edge Diagnosis Technology of Escalator Motor Bearing Based on Deep Transfer Learning. Master’s Thesis.

[13] Pereira R.C.A., da Silva O.S., de Mello Bandeira R.A., dos Santos M., de Souza Rocha C., Castillo C.d.S., Gomes C.F.S., de Moura Pereira D.A., Muradas F.M. (2023). Evaluation of Smart Sensors for Subway Electric Motor Escalators through Ahp-Gaussian Method. Sensors.

[14] You F., Wang D. Escalator Fault Diagnosis Method Based on Svm and Feature Frequency Extraction. Proceedings of the 2022 34th Chinese Control and Decision Conference (CCDC).

[15] Bi X. (2024). Classification and Cause Analysis of Common Faults of Escalators. China Elavator.

[16] Wang Z. (2024). Research and Application of Fault Monitoring and Intelligent Diagnosis Technology of Metro Escalator. Master’s Thesis.

[17] He C., Zheng B., Shi X. Research of Rail Impact of Escalator Based on Residual Peak Envelope Method. Proceedings of the 2023 6th International Conference on Artificial Intelligence and Big Data (ICAIBD).

[18] Zhang X. Research on Key Technologies for Intelligent Analysis of Escalator Risk Management in Large Transportation Hub. Proceedings of the 2024 International Conference on Urban Construction and Transportation (UCT 2024).

[19] Chen Z., Zhang H., Xu X., Liu W., She K., Qiu B. Review of Fault Modes and Fault Diagnosis Methods for Elevator Systems. https://papers.ssrn.com/sol3/papers.cfm?abstract_id=4913944.

[20] Zhang P. (2022). Research on Escalator Performance Monitoring and Cause Investigation Based on Vibration Signal. Doctoral Dissertation.

[21] Chen J., Qi Z., Yang N., Luo W. Development and Application of Vibration Comfort Detection Device for Escalator Based on Mems Acceleration Sensor. Proceedings of the 2022 2nd International Conference on Measurement Control and Instrumentation (MCAI 2022).

[22] Song L. (2023). Research on Health Prediction of Rotating Machinery Based on Deep Learning. Doctoral Thesis.

[23] Jiang X.Y., Yuan H.B., Zhang Z.Z., Chen B.Y., Tong Y.F. Design of Internet of Things-Based Escalator Condition Monitoring and Early Warning System. Proceedings of the 2024 International Conference on Industrial IoT, Big Data and Supply Chain (IIoTBDSC).

[24] Qian S., Ke Z., Huang W., Liu N., Guo W., Gu Q., Wu H. (2023). Research on Failure Diagnosing by the escalator vibration signal with FFT algorithm. Autom. Instrum..

[25] Han P., He C., Lu S. (2022). Bearing incipient fault diagnosis based on VMD and enhanced envelope spectrum. J. Mech. Electr. Eng..

[26] Zhang Y. Analysis of Escalator Based on Spectral Kurtosis and Envelope Demodulation Method Diagnosis and Application of Motor Bearing Faults. Proceedings of the 2023 2nd International Conference on Automation, Robotics and Computer Engineering (ICARCE).

[27] You F., Wang D., Li G., Chen C. (2022). Fault Diagnosis Method of Escalator Step System Based on Vibration Signal Analysis. Int. J. Control Autom. Syst..

[28] Wang W. (2023). Escalator Foundation Bolt Loosening Fault Recognition Based on Empirical Wavelet Transform and Multi-Scale Gray-Gradient Co-Occurrence Matrix. Sensors.

[29] Duan Y., Huang M., Wang S., Liu Y. (2024). Research on escalator bearing fault diagnosis method based on CNN-Transformer model. Mod. Electron. Tech..

[30] Tan B., Huang M., Liu Y., An Q. (2023). Software design of escalator monitoring based on CNN-LSTM fault diagnosis method. Electr. Meas. Technol..

[31] Cao J., Yu Y., Wang Q., Dong Y. (2024). Research on intelligent diagnosis of motor bearing faults based on optimized VMD-CNN-BiLSTM. Mod. Electron. Tech..

[32] Jiang X., Zhang Z., Yuan H., He J., Tong Y. (2025). DAE-Bilstm Model for Accurate Diagnosis of Bearing Faults in Escalator Principal Drive Systems. Processes.

[33] Wang Z., Li S., Xuan J., Shi T. (2024). Biologically Inspired Compound Defect Detection Using a Spiking Neural Network with Continuous Time–Frequency Gradients. Adv. Eng. Inform..

[34] Simonyan K., Zisserman A. (2014). Very Deep Convolutional Networks for Large-Scale Image Recognition. arXiv.

[35] He K., Zhang X., Ren S., Sun J. Deep Residual Learning for Image Recognition. Proceedings of the IEEE Conference on Computer Vision and Pattern Recognition.

[36] Xie S., Girshick R., Dollár P., Tu Z., He K. Aggregated Residual Transformations for Deep Neural Networks. Proceedings of the IEEE Conference on Computer Vision and Pattern Recognition.

[37] Vaswani A., Shazeer N., Parmar N., Uszkoreit J., Jones L., Gomez A.N., Łukasz K., Polosukhin I. (2017). Attention Is All You Need. Adv. Neural Inf. Process. Syst..

[38] Li P., Pei Y., Li J. (2023). A Comprehensive Survey on Design and Application of Autoencoder in Deep Learning. Appl. Soft Comput..

[39] Al Najjar Y. (2024). Comparative Analysis of Image Quality Assessment Metrics: Mse, Psnr, Ssim and Fsim. Int. J. Sci. Res..

[40] Rengasamy D., Jafari M., Rothwell B., Chen X., Figueredo G.P. (2020). Deep Learning with Dynamically Weighted Loss Function for Sensor-Based Prognostics and Health Management. Sensors.

[41] Queiroz S., Vilela J.P., Ng B.K.K., Lam C.T., Monteiro E. (2025). Fast Computation of the Discrete Fourier Transform Rectangular Index Coefficients. arXiv.

[42] Wang J., Yu L., Tian S. (2025). Cross-Attention Interaction Learning Network for Multi-Model Image Fusion Via Transformer. Eng. Appl. Artif. Intell..

[43] Eraliev O., Lee K.H., Lee C.H. (2022). Vibration-Based Loosening Detection of a Multi-Bolt Structure Using Machine Learning Algorithms. Sensors.

[44] Hao J., Shi S., Zhang Y., Li X., Ren X., Gao J., Liu S., Zhang H., Wu J., Zhang B. (2025). A High Signal-to-Noise Ratio Nir Fluorescent Probe for Viscosity Detection in Nafld Diagnosis. Sens. Actuators B Chem..

[45] Zhang W., Peng G., Li C., Chen Y., Zhang Z. (2017). A New Deep Learning Model for Fault Diagnosis with Good Anti-Noise and Domain Adaptation Ability on Raw Vibration Signals. Sensors.

[46] Wang H., Liu Z., Peng D., Qin Y. (2019). Understanding and Learning Discriminant Features Based on Multiattention 1dcnn for Wheelset Bearing Fault Diagnosis. IEEE Trans. Ind. Inform..

[47] Lai H., Huang T., Lu B., Zhang S., Xiaog R. (2025). Silhouette Coefficient-Based Weighting K-Means Algorithm. Neural Comput. Appl..

